# New trends in diagnosing and treating ovarian cancer using nanotechnology

**DOI:** 10.3389/fbioe.2023.1160985

**Published:** 2023-04-04

**Authors:** Juan Zhang, Haigang Ding, Feng Zhang, Yan Xu, Wenqing Liang, Liping Huang

**Affiliations:** ^1^ Department of Gynecology, Shaoxing Maternity and Child Healthcare Hospital, Shaoxing, China; ^2^ Obstetrics and Gynecology Hospital of Shaoxing University, Shaoxing, China; ^3^ Intensive Care Unit, Zhoushan Hospital of Traditional Chinese Medicine Affiliated to Zhejiang Chinese Medical University, Zhoushan, China; ^4^ Medical Research Center, Zhoushan Hospital of Traditional Chinese Medicine Affiliated to Zhejiang Chinese Medical University, Zhoushan, China; ^5^ Department of Medical Oncology, Zhoushan Hospital of Traditional Chinese Medicine Affiliated to Zhejiang Chinese Medical University, Zhoushan, China

**Keywords:** ovarian cancer, nanocarriers, diagnosing, treatment, nanotechnology

## Abstract

Ovarian cancer stands as the fifth most prevalent cancer among women, causing more mortalities than any other disease of the female reproductive system. There are numerous histological subtypes of ovarian cancer, each of which has distinct clinical characteristics, risk factors, cell origins, molecular compositions, and therapeutic options. Typically, it is identified at a late stage, and there is no efficient screening method. Standard therapies for newly diagnosed cancer are cytoreductive surgery and platinum-based chemotherapy. The difficulties of traditional therapeutic procedures encourage researchers to search for other approaches, such as nanotechnology. Due to the unique characteristics of matter at the nanoscale, nanomedicine has emerged as a potent tool for creating novel drug carriers that are more effective and have fewer adverse effects than traditional treatments. Nanocarriers including liposomes, dendrimers, polymer nanoparticles, and polymer micelles have unique properties in surface chemistry, morphology, and mechanism of action that can distinguish between malignant and normal cells, paving the way for targeted drug delivery. In contrast to their non-functionalized counterparts, the development of functionalized nano-formulations with specific ligands permits selective targeting of ovarian cancers and ultimately increases the therapeutic potential. This review focuses on the application of various nanomaterials to the treatment and diagnosis of ovarian cancer, their advantages over conventional treatment methods, and the effective role of controlled drug delivery systems in the therapy of ovarian cancer.

## 1 Introduction

Ovarian cancer (OC) is a prevalent female reproductive organ malignant tumor that is frequently detected at an advanced stage and is prone to spreading to the pelvic and abdominal cavities, causing malignant ascites (Gao et al., 2019a; Lheureux et al., 2019; Kuroki and Guntupalli, 2020). Once recognized as a single entity, ovarian cancer can now be separated into distinct histological subtypes with distinct risk factors, molecular compositions, cell of origin, clinical characteristics, and therapies. Serous, clear-cell, endometrioid, and mucinous carcinomas are some of the epithelial malignancies that make up about 90% of ovarian cancers among these histological subtypes (De Leo et al., 2021). Other uncommon histologies comprise small cell carcinoma (aggressive cancer that mostly affects younger women, with a median age of 25 at diagnosis) with undetermined tissue origin and carcinosarcoma (Callegaro-Filho et al., 2016; Witkowski et al., 2016). About 10% of ovarian cancers are classified as non-epithelial cancers, which include germ-cell tumors and stromal tumors of the sex-cord. Despite recent advancements in diagnosis and therapy, the 5-year survival rate for OC is about 25%–30%, the lowest of all gynecological cancers (Li et al., 2019). Most significantly, metastasis of tumor cells and peritoneal infiltration generate malignant ascites of OC, which has a significant negative impact on patients' quality of life and is one of the leading causes of patient death (Kipps et al., 2013). Advanced OC is mostly treated with surgery and platinum-based chemotherapy. Intraperitoneal chemotherapy has the potential to enhance cytotoxicity and reduce the development of ascites in advanced OC by increasing the tumor’s exposure to antineoplastic agents over the past few decades (Markman, 2003). Platinum-based drugs (oxaliplatin, cisplatin, carboplatin), paclitaxel and mitomycin are widely utilized in both clinical and experimental conditions for intraperitoneal infusion (Glockzin et al., 2013; Wright et al., 2015).

However, one of the negative effects of chemotherapeutics is the destruction of normal cells, which has an adverse impact on the immune system. Most of the chemotherapeutic drugs show their therapeutic action by intervening the cell division of rapidly growing cells. Excessive application and administration of such drugs to healthy tissues and vulnerable areas of the body is the primary factor contributing to such severe adverse side effects (Sinha et al., 2006). In addition, most anticancer drugs have poor bioavailability because of their less aqueous solubility, poor physicochemical properties or overall electronegative surface charge, which prevents the drugs from penetrating the cells because of the cytomembrane’s negative charge. This, in turn, leads to poor cell adhesion as the cell membranes’ innate negative charge repel such drugs and, ultimately, poor bioavailability (Tian et al., 2016). This encourages medical professionals to prescribe a higher drug dose than is necessary to maintain diffusion-controlled phenomena. Current methods of diagnosis and therapy are insufficiently sensitive and effective to identify and treat OC at an early stage. In addition, the high expenses and lack of a defined detection point lead to a delayed diagnosis. Nanotechnology is contributing an important role in the therapy and diagnosis of OC. The primary objective of nanotechnology is to discover new ways and strategies for treating numerous diseases, such as OC (Satpathy et al., 2019; Talluri and Malla, 2019). The benefits of nanocarrier applications include targeted administration of hydrophobic compounds, delivery carrier stabilization, lowering of antineoplastic drugs' systemic toxicity, and enhanced biodistribution and pharmacokinetics (Seca et al., 2019). Contrasting agents [iron oxide, magnetic, and gold nanoparticles (NPs)] and fluorescent agents (quantum dots) are among the nanocarriers utilized for imaging or diagnostics; These agents may be loaded with targeting moieties (Kotcherlakota et al., 2017; Yao et al., 2018). Some nanocarriers (such as carbon nanotubes, magnetic, and gold NPs) have inherent optical characteristics including fluorescence and Raman scattering, rendering them advantageous for optical imaging and sensing applications. Several fluorescent methods for imaging OC using quantum dots were evaluated in mice both *in vitro* and *in vivo* (Baghbani and Moztarzadeh, 2017).

Various types of OC nanocarriers have been discussed in this review, with a particular emphasis on their usage in targeted therapy and diagnosis. The therapeutic potential and preferred targeting of OC through the development of ligand-functionalized nanoformulations have been studied and compared to those of non-functionalized nanoformulations. Moreover, nanomaterial-based tumor markers biosensors for the diagnosis of OC are described.

## 2 Challenges in OC detection and therapy

**FIGURE 1:**
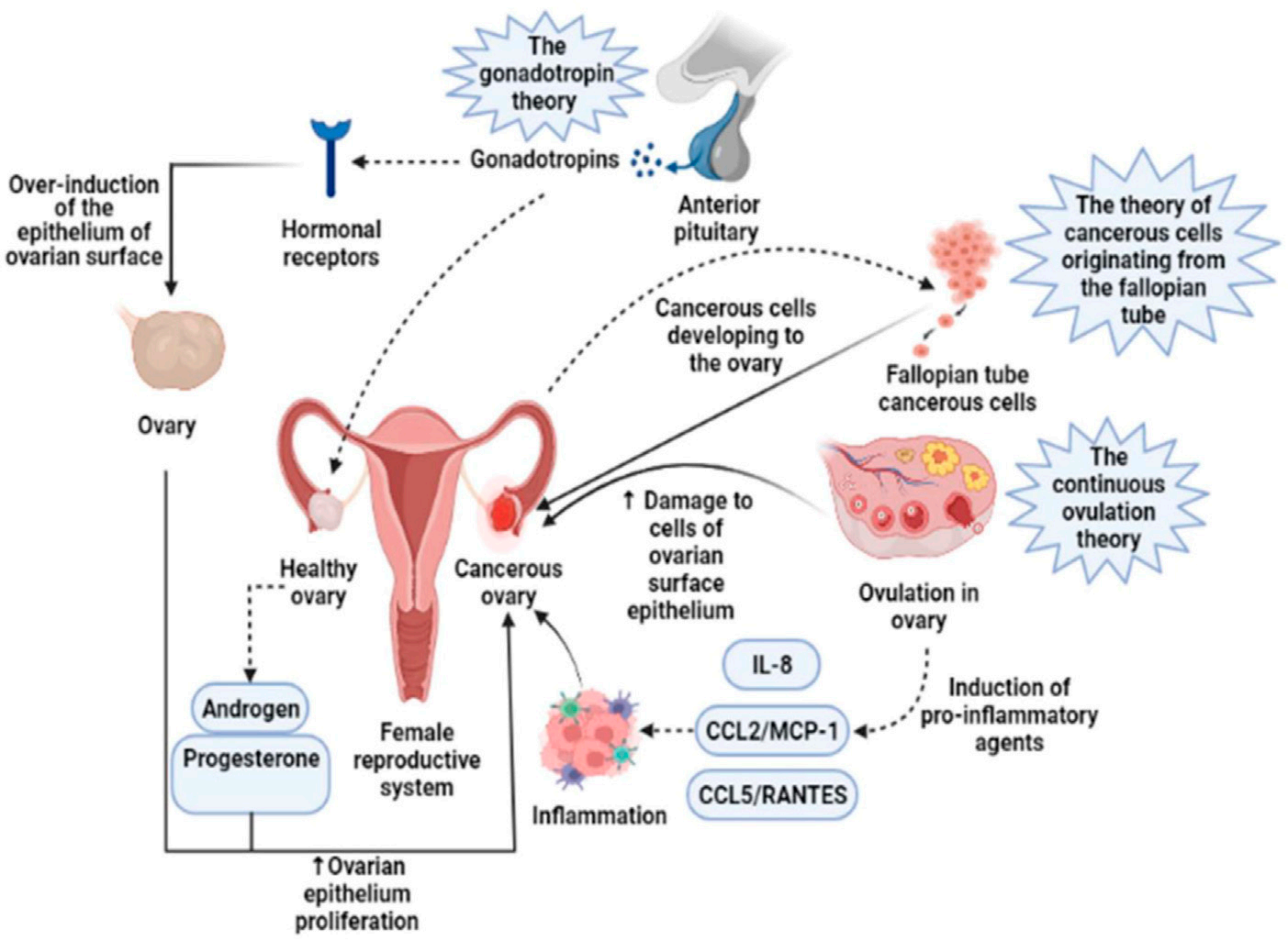
Three basic theories concerning the onset of OC are based on the stimulation of the ovarian surface epithelium by hormone receptors, the enhanced production of pro-inflammatory substances during continuous ovulation, and malignant cells arising from the fallopian tube. IL-8, Interleukin-8; CCL2/MCP-1, Monocyte chemoattractant protein-1; CCL5/RANTES, CC Chemokine Ligand-5 (Rezaei-Tazangi et al., 2021).

There are three basic theories related to the onset of OC including the stimulation of the ovarian surface epithelium by hormone receptors, the enhanced production of pro-inflammatory substances during continuous ovulation, and malignant cells arising from the fallopian tube (Figure 1). About 90% of ovarian malignancies are epithelial OCs that develop from the ovarian surface epithelium. Ovary and omentum are frequently affected by OC, which also frequently have intraperitoneal metastases and diffuse malignant ascites (Chen et al., 2019). Epithelial OC metastasis entails the shedding of cells from the initial OC as single cells or as multicellular/spheroids aggregation, which later interact with mesothelial cells lining the peritoneum’s inner surface and disseminate to neighboring pelvic organs (Lengyel, 2010). About 75% of patients with OC had an intraabdominal illness at the time of diagnosis, and only about 40% of patients in stage 3 survive for 5 years after diagnosis (Giampaolino et al., 2019). Numerous patients experience relapses of OC metastases, which are primarily restricted to the peritoneum. However, intraperitoneal (IP) administration of chemotherapy is linked to significant toxicity (Jayde et al., 2010; Joyce Liu and Matulonis, 2010). Radiation therapy and chemotherapy are not effective on spheroids or multicellular aggregates, which may cause a relapse during treatment (Shield et al., 2009). Emerging evidence reveals that the newly found OC stem cells are particularly resistant to standard cytotoxic chemotherapeutic drugs and are capable of generating and propagating *in vitro* as spheroids/multicellular aggregates (Fong and Kakar, 2010; Hu et al., 2010). Current evidence suggests that OC cells are very resistant to traditional chemotherapeutics, which in turn contributes to the disease’s recurrence and development of resistance to treatment (Paffenholz et al., 2022). OC is often misdiagnosed as a gastrointestinal disorder, and early detection is missed because of a lack of reliable screening and detection technologies.

Radiation treatment and chemotherapy are currently being tested in clinical studies for patients with early-stage OC to improve their chances of survival. Surgery with a diameter larger than 1 cm is the primary line of treatment for cancer patients in the advanced stages. This is followed by IP or intravenous (IV) administration of active drugs containing platinum, such as cisplatin and paclitaxel. Clinical trials comparing IP cisplatin to IV cisplatin were regulated by the Gynecology Oncology group. According to the findings of the following study, intraperitoneal (IP) cisplatin therapy can prolong intravenous (IV) chemotherapy for a longer period (Krivak et al., 2008). Generally, poor treatment outcomes and a high rate of relapse were the results of all treatment methods, necessitating more attempts to establish a more effective therapeutic regimen for patients with OC. The main cause of the rise in OC patient mortality is the high death rate brought on by late diagnosis, which correlates to a high rate of proliferative activity within the abdominal cavity. OC can be detected early with the help of physical diagnosis, clinical history, detection of CA-125 serum protein, ultrasound evaluation, and physical examination, all of which will be made easier with the help of modern technologies (Chen et al., 2019; Stewart et al., 2019). More than 80% of OC patients had high serum CA-125 levels. In addition to being an effective indicator for OC diagnosis, a rise in CA-125 levels indicates ineffective treatment (Chang et al., 2019). Additionally, lysophosphatidic acid (LPA) is a marker for the diagnosis of OC in women and women with benign gynecologic disorders. The LPA level may be more useful than the CA-125 level in diagnosing OC at an early stage (Cao et al., 2015).

## 3 Advantages of nanotechnology in OC diagnosis and treatment

**FIGURE 2:**
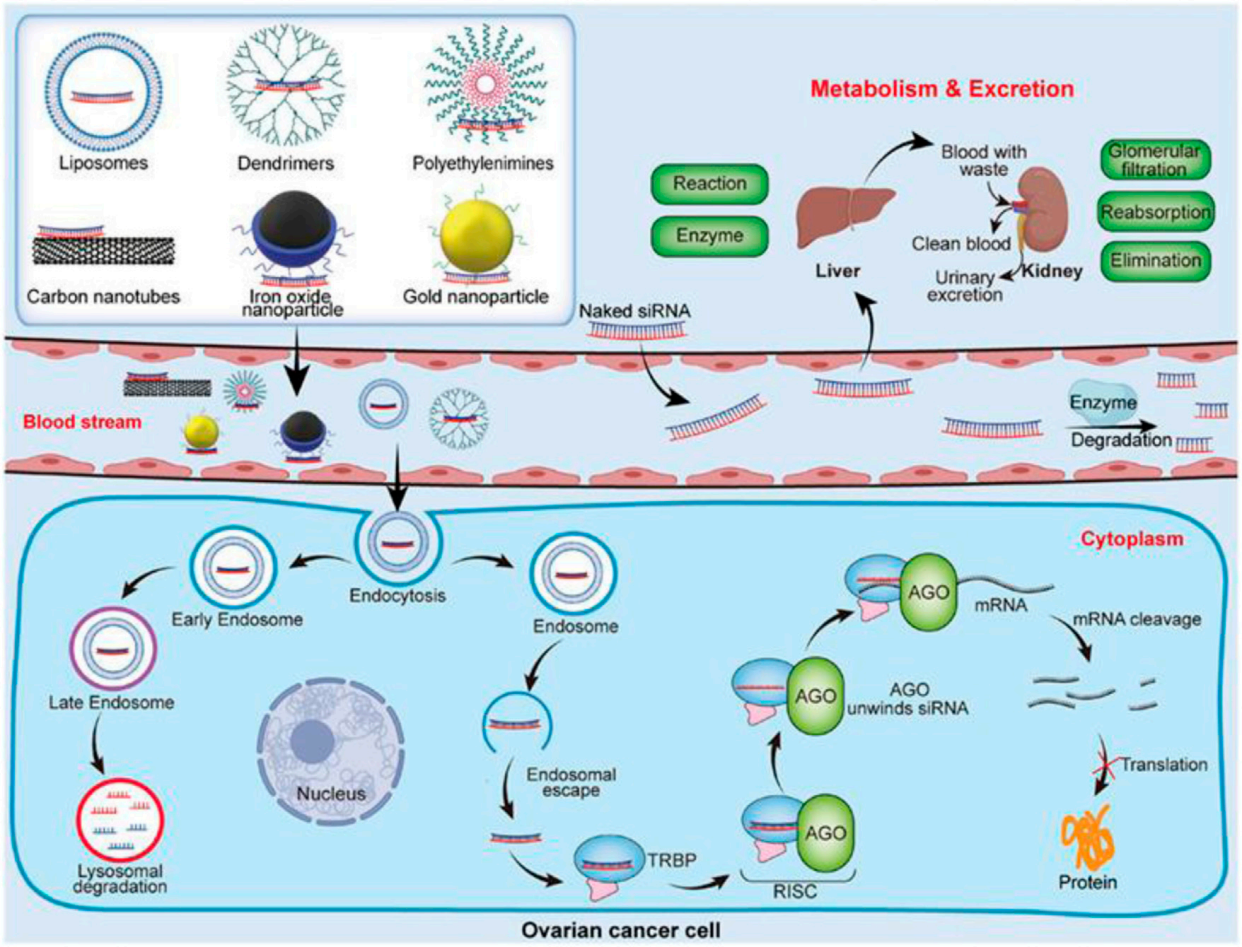
Nanocarrier-mediated siRNA delivery and its mechanism of action. Reproduced with permission from reference (Zou et al., 2021).

OC does not typically exhibit any clinical symptoms in its early stages, which makes it difficult to diagnose this tumor. The symptoms of OC in its early stages are commonly misdiagnosed as those of other benign pathologies, including urogenital infections, gastrointestinal disorders, and benign ovarian lesions (including ovarian cysts, fibromas, and teratomas) (Goff, 2012). The utilization of positron emission tomography, computed tomography, magnetic resonance imaging, real-time elastography, and ultrasound are remarkable among the earliest cancer diagnostic technologies (Kong et al., 2019). Nanotechnology provides considerable potential for addressing current challenges and enhancing OC diagnostics and therapy. Although the nanomedical field remains in its early phases, there has recently been a rise in research in this area, notably concerning applications for OC. The benefits of utilizing nanomaterial-based biosensors to deal with these problems include improved sensitivity and selectivity (Yang et al., 2022). Multiple advantages are associated with magneto-resistive, electrical, and electrochemical sensors (Mohankumar et al., 2021). Finally, investigations on portable sensors for use outside of the clinic in identifying OC biomarkers are still quite limited (Sohrabi et al., 2022). This biosensing platform, which is currently in its early stages, must be integrated at the systems level to commercialize the biosensors. There is great promise in ongoing efforts to develop low-cost, reusable biosensors based on paper-based components, flexible field effect transistors, large magnetoresistive elements, or microfluidic lab-on-chip platforms for detecting OC biomarkers. With the advancement of nanotechnology, promising technologies are anticipated in the near future for scaled-down sensing processes and analysis kits, which would pave a new approach for OC patients to monitor their health with ease. A possible treatment strategy based on the nanocarrier-mediated siRNA delivery and its mechanism of action for OC has been depicted in Figure 2 **.**

The Food and Drug Administration (FDA) of the United States has previously approved a variety of nanoparticle-based medications for targeted cancer therapy. For instance, the FDA has approved the use of Onivyde, a PEGylated Liposomal Carrier for Irinotecan, to treat Metastatic Pancreatic Adenocarcinoma. Irinotecan, which inhibits topoisomerase I, was found to accumulate in tumors, where it could be released slowly and exert its anti-tumor effects for a longer period of time (Passero et al., 2016; Zhang, 2016). Similarly, the use of Doxil as a cancer treatment demonstrates the significance of nanotherapy in OC. Doxil is a liposomal formulation of the drug doxorubicin HCl. The drug’s liposome encapsulation prolongs drug circulation time, slows down the drug’s removal from the blood, and encourages drug exposure in tumor cells (Green and Rose, 2006). Palliative therapy for cancer patients has been added to the applications of nanomedicine for cancer. For example, Eligard, a leuprolide acetate polymeric nanoparticle, is used as a palliative treatment for patients with advanced prostate cancer by preventing the release of gonadotropin-releasing hormone (Enayati et al., 2017). Due to its superior optical properties and less invasive penetration modes, nanotechnology also used to create nanostructures for protein tracking, *in vivo* imaging, intracellular transport trafficking, and other critical mechanisms (Peñaloza et al., 2017; Sarathkumar et al., 2021). In a current phase II clinical trial study, silica NPs are being used to track nodal metastases and view lymph nodes in real-time in patients with head and neck cancer undergoing surgery (Bradbury et al., 2016). Because of this, it is clear that similar approaches are needed for both the diagnosis and treatment of OC. In this section, various targeting approaches for drug delivery systems based on nanomaterials are examined.

## 4 Current screening of OC

Screening for OC currently available in clinical practice is limited to clinician-performed physical examinations, transvaginal ultrasound (TVU) imaging of the adnexa, and measurements of the protein biomarker cancer antigen 125 (CA125) levels in the serum. The most frequent imaging technique used to find OC is TVU, which enables medical professionals to spot changes in ovarian tissues' size and shape. Radiologists examine transvaginal ultrasound images to look for certain clinical features in accordance with the International Ovarian Tumor Analysis’s simple rules. Characteristics such as ascites, papillary projections, and internal blood flow are evaluated to make predictions about the presence and development stage of malignant masses (Kaijser et al., 2013).

The most common OC biomarker, CA125, is a glycoprotein found on the surface of epithelial cells and is thought to encourage the growth and metastasis of cancer cells. 80% of OC patients were found to have elevated serum levels (>35 U/mL) of CA125 (Zurawski et al., 1988). Unfortunately, additional studies into the therapeutic benefit of CA125 screening have not yet shown a significant benefit to patients, primarily because early disease stages lack clinical sensitivity for CA125. When used in conjunction with multimodal diagnostic techniques, preoperative CA125 levels have been shown to be of limited value or better discrimination of ovarian masses (Timmerman et al., 2007). The results of an OC screening trial revealed that CA125 screening had a positive predictive value of only 4%, which could be increased to 26.5% by adding transvaginal ultrasound. However, a 15-year follow-up has not yet demonstrated that this combination significantly improves patients' survival rates (Buys et al., 2005; Pinsky et al., 2017). Furthermore, only 2% of the 28,506 women who participated in that screening trial and received results for both the transvaginal ultrasound and CA125 had abnormalities in both tests (Buys et al., 2005). OCs of the endometroid, mucinous, and other less common subtypes were found to express CA125 at lower levels than serous carcinomas, demonstrating the inadequacy of CA125 as a sole biomarker for the diagnosis of OC (Høgdall et al., 2007). Furthermore, irrespective of OC, epidemiological factors like age, ethnicity, race, and obesity were discovered to be connected to influencing CA125 serum levels (Pauler et al., 2001). Despite its shortcomings as a screening biomarker, CA125 has been shown to be clinically useful for patients when used post-operatively to monitor therapeutic efficacy and aid in the detection of recurrent disease (Piatek et al., 2021).

Cancer antigen 19-9 (CA19-9) and human epididymis protein 4 (HE4) are two additional single biomarkers that have recently drawn attention due to their increased expression in less prevalent subtypes of OC. In contrast to mucinous or clear-cell tumors, endometroid and serous ovarian tumors have been reported to be associated with increased secretion of HE4, which is a surface glycoprotein similar to CA125 (Ruggeri et al., 2011). According to studies comparing HE4 and CA125, HE4 changes occur two to 3 months earlier than CA125 (Abbink et al., 2018). HE4 was found to be comparable to CA125 in monitoring disease progression, so in 2008 the FDA approved HE4 serum tests for use in following women who were already diagnosed with epithelial OC (US Food and Drug Administration, 2023). It is critical to note that while HE4 and CA125 are FDA-cleared for recurrence monitoring, neither biomarker is FDA-cleared to be utilized as a preoperative diagnostic and should not be used without additional imaging or medical evaluation. Increased expression of the monosialoganglioside CA19-9 has been associated with several gastrointestinal tract tumors with mucinous histology, including those of the pancreas, gall bladder, and liver (Motoyama et al., 1990). Although CA19-9 overexpression has been reported in primary mucinous ovarian carcinomas in other studies, there is conflicting evidence regarding the preoperative use of this biomarker in distinguishing malignant from benign mucinous ovarian tumors (Kelly et al., 2010; Lertkhachonsuk et al., 2020).

## 5 Nanotechnology approaches for diagnosis of OC

**FIGURE 3:**
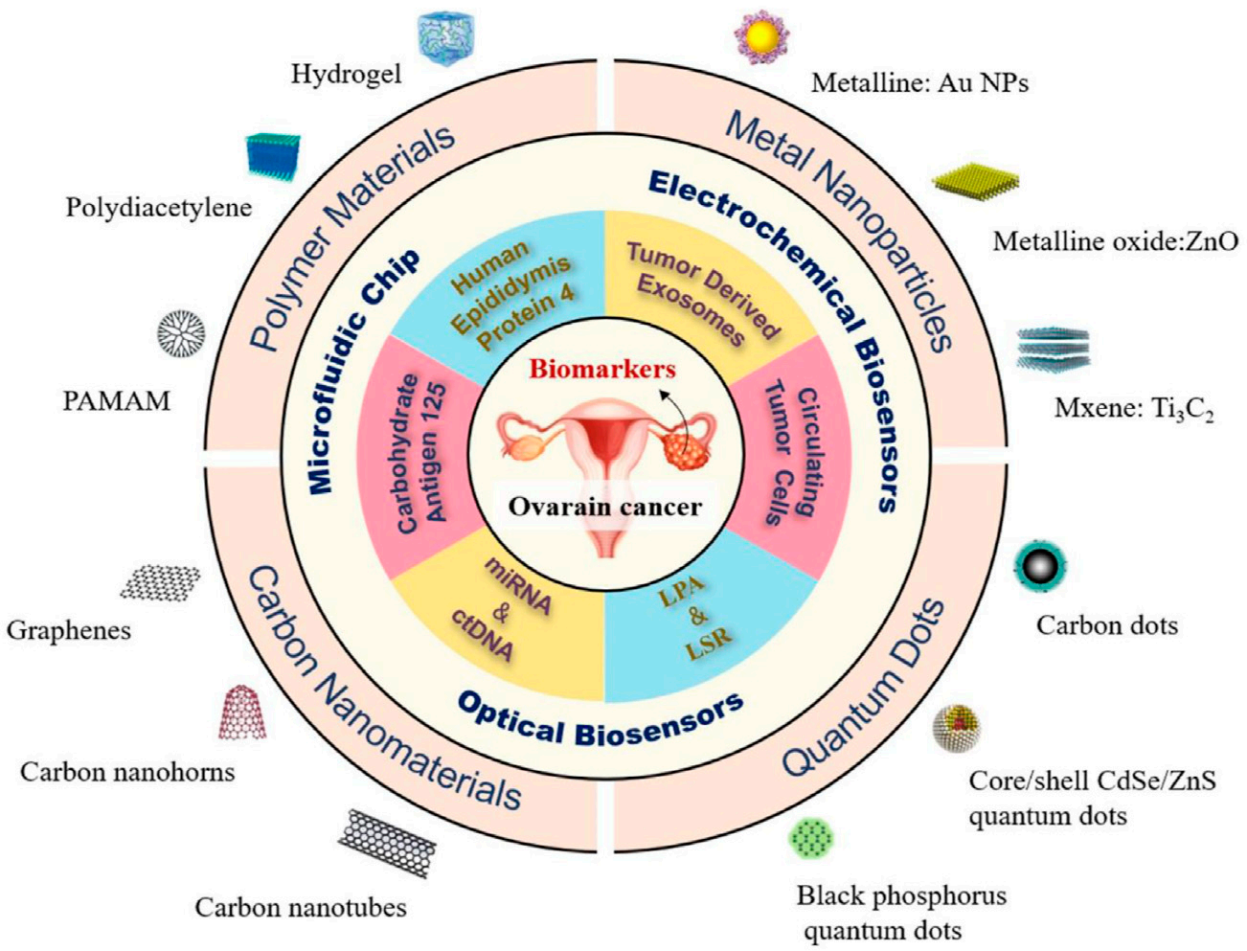
The OC biomarkers detection with different strategies including optical biosensors, microfluidic chips, and electrochemical biosensors, in combination with nanomaterials, including carbon nanomaterials, quantum dots (QDs), polymer materials, and metal NPs (Yang et al., 2022).

Nanotechnology is a relatively new field that can be defined as the practical application of nanoscience that results in a process or a product based on a single or multiple integrated nanoscale components with at least one dimension in the range of 1–100 nm. Imaging agents, drugs, targeting molecules like antibodies or ligands, and polyethylene glycol (PEG), which prolongs the half-life of therapeutic agents and encourages passive and active tumor targeting, can all be loaded onto nanocarriers through chemical conjugations or physical adsorption (Chen et al., 2022). Examples of nanocarriers include self-assembled polymers, liposomes, micelles, dendrimers, hydrogels, magnetic NPs, quantum dots, carbon-based nanocarriers (carbon nanotubes, and bucky balls), and oxide or metal-based NPs (silica, colloidal gold, and titanium dioxide). One of the objectives of nanotechnology is to enhance the methodologies used for therapy, diagnosis, or combining the two (theranostics) in a variety of diseases, such as OC. The stage of OC at the time of diagnosis has a significant impact on the survival rate of patients. *In vitro* diagnosis (IVD) of OC-related biomarkers has become a significant advancement in the diagnosis of OC. Emerging biomarkers (CTC, TEX, LPA, metabolites, LSR, ctDNA, and miRNA) and classic biomarkers (HE4 and CA125) show considerable potential. The detection performance of these biomarkers is enhanced by a variety of superior detection technologies, including optical biosensors, microfluidic chips, and electrochemical biosensors, in combination with nanomaterials, including carbon nanomaterials, quantum dots (QDs), polymer materials, and metal NPs **(** Figure 3 **)** (Yang et al., 2022).

### 5.1 Electrochemical nanosensors

OC biomarkers can be detected quickly and accurately with electrochemical nanosensors in blood, urine, and saliva due to the sensitivity of the technology (Manasa et al., 2022). Furthermore, the incorporation of NPs with electrochemical systems (such as magnetic NPs, gold NPs, and carbon nanotubes) offers highly sensitive enhancement and multipathing capabilities for locating and managing cancer biomarkers (Mohammadpour-Haratbar et al., 2022).

Electrochemical nanosensors have demonstrated promising methodologies and roles for accurate biomarker detection in OC, and they typically have the detection capacity for biomarkers in minute amounts and other analytes (Parmin et al., 2021). Electrochemical sensors with NPs that provide multipathing capabilities and increased sensitivity were employed for the detection and identification of OC biomarkers (Barani et al., 2021). Current diagnostic methods have led to the detection of CA-125, a biomarker in OC stages I and II, in 75% of patients. Improved early detection and diagnosis may be possible with the monitoring of CA-125 indicators at low concentrations (Samborski et al., 2022). Similar to this, graphene nanosensors for CA-125 biomarker detection were created by a research team to provide label-free detection after polyaniline surface precipitation and coupling with anti-CA 125 antibodies. The developed nanosensor, with a 0.92 ng/μL detection limit, was the most sensitive detection technique for CA-125 at the time of technological advancement (Gazze et al., 2018). E-cadherin was used as a tumor detection biomarker among others since its expression was discovered to be negatively correlated with the presentation and identification of OC (Rea et al., 2018). Carbon nanotube and Quantum dot nanocomposites were employed as electrochemical nanosensors based on the detection of e-cadherin alterations in a study by Du et al. to identify OC biomarkers. The electrochemical nanosensor’s results for low E-cadherin detection as an OC biomarker demonstrated responsive and quick electrochemical signal transduction, which was attributable to the synergistic effect of applied nanomaterials (Du et al., 2020).

### 5.2 Optical nanosensors

Optical biosensors have biorecognition sensing capabilities due to the incorporated optical transducer system, making it a flexible analysis tool. An optical biosensor’s main objective is to transmit a signal that is directly proportional to a selected agent or biomarker (Sohrabi et al., 2020). There are several options for optical biosensors that have been developed, and they can be categorized as optical biosensors based on fluorescence, chemiluminescence, surface plasmon resonance, and electrochemiluminescence.

For the detection of multiple cancer biomarkers in specimens from cancer patients and the detection of specific biomarkers *in-situ* utilizing implantable sensors, Yaari et al. (2022) suggested two platforms. These technologies utilized the distinctive optical properties of sensors and artificial intelligence algorithms based on single-walled carbon nanotubes. The sensors used a library of single-stranded DNA sequences and carbon nanotubes with different chiralities to identify biomarkers in clinical samples. A sensor that can be implanted was created to serve as an implantable intrauterine device to detect biomarkers inside the uteri of the patients. The sensor could identify benign and malignant cases and detect cancer biomarkers. A single-marker immunoassay may produce unreliable results when employed in the cancer clinical diagnosis. It is preferable to have a reliable and accurate method for measuring multiple tumor markers simultaneously. Fluorescence immunosensors have been shown to be effective, as reported by Bahari et al. (2021), for the simultaneous estimation of CA15-3 and CA125, two tumor markers. In addition to the use of magnetic graphene oxide (GO-Fe_3_O_4_) as a support material for molecularly imprinted polymers on the surface, the use of nickel nanoclusters (Ni NCs) and cadmium (Cd) NCs as efficient and cost-effective emitters have also been introduced. The proposed approach for the clinical diagnosis of CA 15-3 and CA 125 tumor markers had excellent characteristics, including a good reproducibility, broad linear range, and ease of operation. In conclusion, optical biosensors were developed through a simple preparation procedure, and they demonstrated quick analysis times and good stability. These findings show enormous potential for the evaluation of CA-125 in real samples and pathological diagnosis, cell lines, and promise as an approachable assay for enhancing point-of-care analysis.

### 5.3 Micro/nanofluidic based nanosensors

In biomedical research or biochemical processes, a liquid droplet’s motion is frequently controlled by microfluidic and nanofluidic devices. The micro/nanofluidic platforms have numerous benefits such as efficient fluid processing, miniaturization, simple integration, rapid analysis, and low sample consumption, therefore offers extensive opportunities for cells analysis (Han et al., 2022). Usually, microfluidic separation of extracellular vesicles and circulating tumor cells depends on physical or affinity characteristics. Size, dielectrophoresis, and hydrodynamics-based isolation are all examples of physical property-based isolation. There are aptamer and antibody-based isolation methods for affinity-based isolation. The micro/nanofluidic systems are integrated with a variety of detection methods after successful isolation, including, colorimetry, fluorescence, electrochemistry, and surface-enhanced Raman scattering (Gao et al., 2019b; Liu et al., 2022).

Dissociating exosomes from OC serum has been reported using an antibody-functionalized microfluidic framework to identify biomarkers present in cancer exosome membranes (e.g., EpCAM, CD9). After being significantly released, the intact exosomes eventually internalized in OC cells (Hisey et al., 2018). To extract exosomes from culture media and clinical specimens, a novel microfluidic system was constructed in which isolation of sample was based on adhesion molecule of epithelial cells and high-precision CD63 expression, thereby avoiding the issue of contamination. The developed microfluidic system assisted in the diagnosis of high-grade serous OC in women (Dorayappan et al., 2019). Personalized medicine techniques employ functional and molecular analysis of a patient’s cancer cells to select therapies with the highest probability of success. Unfortunately, isolation of malignant cells is challenging due to the metastatic nature of such cancers that generally propagate to multiple parts of the body and can not be distinguished easily in multiple cell populations. Therefore, the isolation of cancer cells from these confounding cells is essential for their detection and analysis. To isolate tumor cells from OC patients' ascites fluid without using a label, Stone et al. (2021) designed a microfluidic platform. Cells were separated based on their biomechanical properties without the need for labeling or any other pre-sort interference in this method. The technique is also helpful when the cells of interest lack specific surface markers. The technique was applied to model OC cell lines to distinguish between more invasive and less invasive subtypes with a sixfold enrichment. This technology is believed to enable the application of personalized medicine based on analysis of patient specimens obtained *via* liquid biopsy such as ascites in women with OC, for rapid assessment of the development of metastatic disease and patient-specific treatment determination.

### 5.4 Nanoparticles in imaging of OC

Contrast agents utilized in MRI/CT scans to identify tumors have disadvantages such as toxicity, short retention time, and short imaging time. Imaging with multiple spectral photoacoustics enables spectrally enhanced images that penetrate deeper than conventional contrast agents. In a recent study, the cargo release kinetics of V7-NPs, which were created from mesoporous silica NPs with wormhole pores (V7-RUBY) and loaded with an imaging dye (IR780), were assessed. Both proximal tubule kidney cells and non-malignant hepatocytes were evaluated for V7-RUBY’s toxicity. Utilizing the ovarian tumor’s acidic pH-controlled environment, chitosan was able to regulate slow cargo release from V7-RUBY (Samykutty et al., 2018). A short light pulse is applied to tumor tissues during photoacoustic imaging (PA). A pressure wave that can create an acoustic image is produced as a result of the increased thermal expansion. The production of high-resolution, depth tomographic images by PA has been demonstrated in numerous studies. PA has been combined with endogenous substances like melanin and exogenous substances like NPs for the effective identification of target tissue.

**FIGURE 4:**
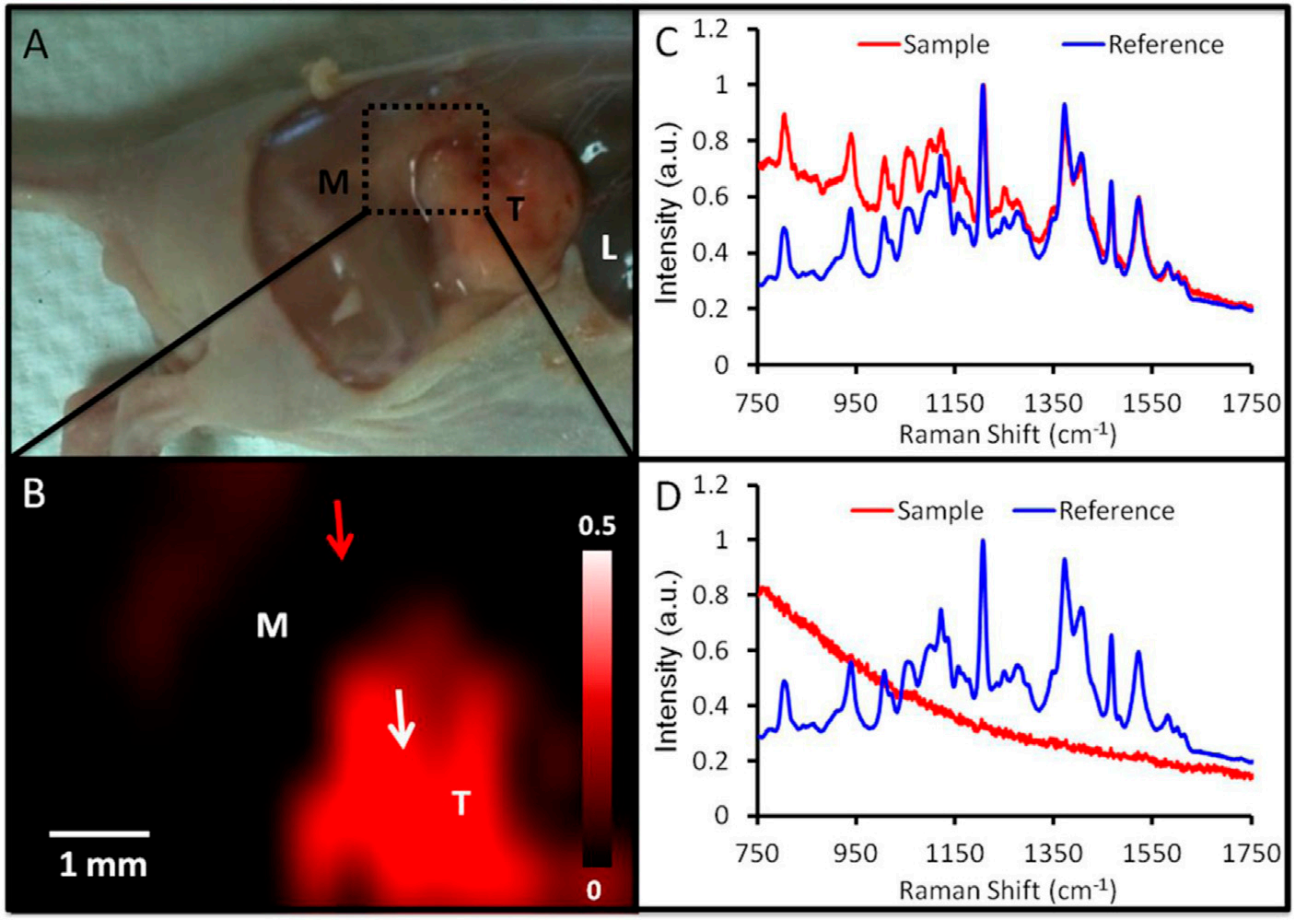
Identification of tumor margins using SERS imaging. **(A)** Image of a mouse carrying a 2008 tumor (T = tumor; M = muscle; L = liver) with the epidermis removed. The region depicted by SERS imaging 24 h after injection is denoted by a green box. **(B)** SERS imaging of the area denoted in panel A reveals an increase in SERS in the tumor (red). This SERS map was produced by correlating the SERS spectra at various spatial locations (red curve in panels C and D) to an *ex vivo* reference spectrum (blue curve in panels C and D). A close match between the reference and sample spectra **(C)** results in an intense pixel on the SERS map (tumor; white arrow, B). A dim pixel results from a poor match between the reference and sample spectra **(D)** (adjacent muscle; red arrow in B). Reproduce with permission frm ACS 2012 (Jokerst et al., 2012).

Surface-enhanced Raman spectroscopy (SERS) is a unique characteristic of noble metal NPs because of their inherent localized surface plasmon resonance. *In vivo* imaging of subcutaneous xenograft models of ovarian tumors was successful using gold nanorods that combined the SERS and PA imaging modalities as shown in Figure 4 (Jokerst et al., 2012). Para-mercaptobenzoic acid-labeled chitosan-gold-silver NPs were employed as SERS nanotags for changes in intracellular pH and cell imaging in a related study (Hada et al., 2019). An anti-folate receptor antibody was coupled to gold and silica shell NPs using a PEG-succinimide-maleimide cross-linker, with IR780 near-infrared dye serving as the imaging agent. Surface-enhanced resonance has been utilized to develop NPs. Raman ratiometric spectroscopy is employed in xenograft ovarian tumor models to promote efficient tissue penetration and tumor imaging (Oseledchyk et al., 2017). The use of magnetic iron oxide NPs (Fe_3_O_4_) as T2 MRI contrast agents has increased recently. Few studies, however, have investigated their potential as OC contrast agents. One such study found that branched PEI-conjugated Fe_3_O_4_ NPs prepared *via* a reduction route and targeted to folic acid were effective in identifying ovarian tumors that were situated in the nude mice abdominal cavity (Zhang et al., 2016).

Short laser pulses are produced in the near-infrared region by non-linear optical imaging using two-photon intense excitation, which results in improved imaging. Due to the development of the fluorescence signal close to the fluorescence spot, this imaging method eliminates the need for a background-free signal and prevents photodamage to living tissue, resulting in higher axial resolution (Juvekar et al., 2022). Researchers used this method to examine the ability of variously shaped gold NPs covered in gelatin to contrast images and their effectiveness as a reliable contrast agent for visualizing NIH: OVCAR-3 cells. Gelatin-coated gold NPs have been identified as potent contrast agents for fluorescence imaging in clinical therapy and diagnosis (Craciun et al., 2021). Due to the limited number of studies performed on image contrast agents based on NPs for the identification of ovarian tumors, further investigation of the imaging-related characteristics of NPs is necessary to identify broader opportunities for imaging tumors.

## 6 Nanotechnology approaches in OC treatment

The standard first-line chemotherapeutic regimen of taxane and platinum-based medications, such as cisplatin or carboplatin with paclitaxel, is used as the primary treatment for OC following cytoreductive surgery (Williams et al., 2007). However, the majority of patients will eventually experience an OC relapse. Due to the limited and variable patient response, the additional chemotherapeutic agents available to treat platinum-resistant cancer cells are ineffective and, at best, only lead to temporary remission (Markman et al., 2004). Biological therapies (also known as molecular therapies or targeted therapies) can be used in conjunction with chemotherapy to improve its efficacy by inhibiting molecular pathways that contribute to carcinogenesis. These pathways include HER2, epidermal growth factor receptor (EGFR), vascular endothelial growth factor (VEGF), and poly (ADP-ribose)-polymerase (Cui et al., 2021; Perez-Fidalgo et al., 2021). Enrolling in a clinical trial is another option for OC treatment at any stage, although it attracts primarily advanced OC patients because it gives patients hope when all other treatment options have been extensively tried and aid in the development of future treatments.

When compared to the direct administration of chemotherapeutic agents, drug delivery *via* nanocarriers offers numerous benefits. These benefits include the following: 1) the delivery of drugs that have a low solubility by either enclosing the drugs within the nanocarriers' hydrophobic interfaces or acting as the carriers for the drugs in the blood; 2) lowering chemotherapeutic agents' systemic toxicity; 3) stabilizing their cargo by increasing the biodistribution and pharmacokinetics of the therapeutic agents while reducing renal clearance and extending circulation time owing to drug encapsulation and protection against inactivation by metabolic enzymes; and 4) overcoming drug resistance by selectively targeting cancer cells during nanocarrier uptake *via* cellular endocytic pathways and simultaneously multiple chemotherapeutic agents are delivered to the tumor while evading the cellular drug efflux pump (Mosleh-Shirazi et al., 2021; Tunç and Aydin, 2022). The most recent findings in nanomedicine that improve OC treatment are presented in the following sections.

### 6.1 Liposomes

Typically, liposomes are composed of one or two lipid bilayers and possess a spherical shape. In general, liposomes are used for the delivery of both hydrophilic and lipophilic drugs, with the lipophilic drug being incorporated into the lipid bilayer and the hydrophilic drug being stabilized within the aqueous core (Minocha and Kumar, 2022). PEGylation is performed to prevent liposomal elimination by the phagocytic system and enhance their circulation. The proliferation of OC cells was inhibited by using PEGylated liposome nanoformulations loaded with paclitaxel and testing them in an *in vitro* and *in vivo* model (Qi et al., 2018). After receiving treatment with a manufactured nanosystem, the OC cells' aggressiveness was significantly reduced. Additionally, the high expression of ERK and caspase 3/9 in OC cells led to the induction of apoptosis.

**FIGURE 5:**
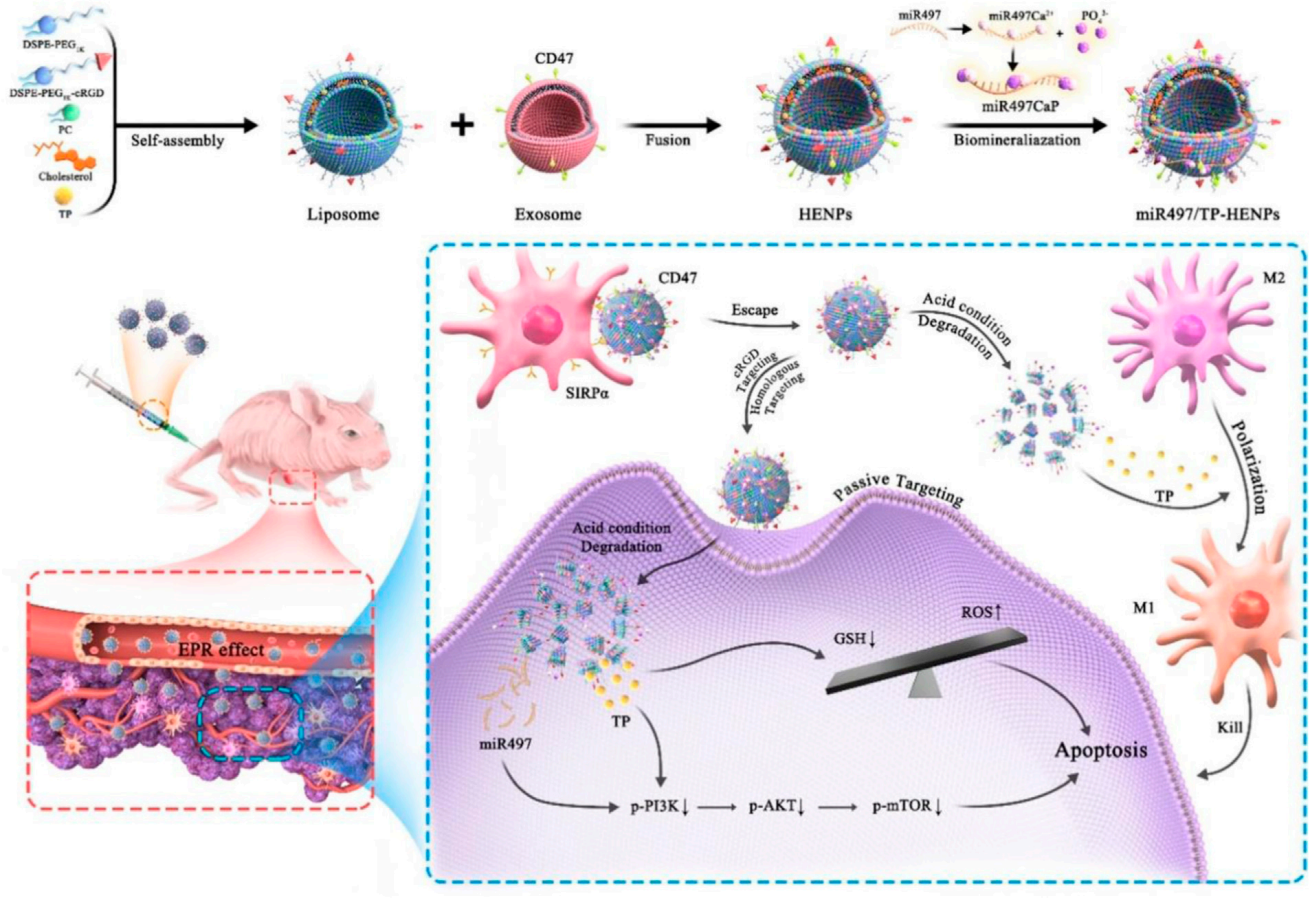
Diagram illustrating the formation process and miR497/TP-HENPs' mechanism of action (Li et al., 2022).

Overexpression of miR497 may be used to overcome OC chemotherapy resistance by inhibiting the mTOR pathway. The PI3K/AKT/mTOR pathway is implicated in tumor chemoresistance. By simultaneously suppressing the mTOR signaling pathway, miR497 and triptolide (TP) could be used in combination to further surmount OC chemoresistance. Li et al., has recently reported an exosome/liposome hybrid nanoparticle system for the co-delivery of miR497 and TP to overcomes chemoresistant ovarian cancer as shown in Figure 5. In OC cells, miR497/TP-HENPs functioned through the following mechanisms: i) The nanoscale size of the nanoplatforms resulted in the enhanced permeability and retention (EPR) effect, ii) The efficiency of NPs' homing targeting was further improved by the use of exosomes and cRGD, iii) CD47 on the surface of exosomes prevented nanoparticle removal by the MPS system, iv) The PI3K/AKT/mTOR pathway was synergistically inhibited by miR497 and TP, v) ROS production was stimulated by TP, and vi) OC resistance was effectively overcome by TP’s modulation of M2 macrophage polarization into M1 macrophages (Li et al., 2022).

When liposomes are functionalized with a ligand, they can target different types of tumor cells and may improve the effectiveness of drug delivery to cancer cells. The liposome with ligand on its surface makes it easier for the active drug to target receptor sites and to directly uptake the drug into tumor cells without having any negative effects on normal cells (Belfiore et al., 2018). Doxorubicin-loaded pegylated liposomes decorated with transferrin and octa-arginine were created by Deshpande and his research team. The formulation was tested in A2780 OC cells that had overexpressed transferrin receptors (TfRs). By inhibiting R8 (macropinocytosis) and Tf (receptor-mediated endocytosis, RME) cell uptake, the modified liposome dramatically improves cytotoxicity *in vitro* and therapeutic efficacy *in vivo* (Deshpande et al., 2018). Using a combination of exosomes and liposomes, Li et al. (2022) developed a novel nano-system for the codelivery of triptolide and miR497 in the treatment of OC that is resistant to standard chemotherapy. CD47-expressing tumor exosomes were fused with cRGD-modified liposomes containing triptolide and miR497 to create hybrid NPs. The uptake of the NPs by tumor cells was demonstrated *in vitro*, and this uptake resulted in a significant increase in the apoptosis of tumor cells. Similarly, the tumor microenvironment was enriched with hybrid NPs, which then displayed potent anticancer activity without causing any unwanted side effects *in vivo*. They increased the production of reactive oxygen species (ROS), dephosphorylated the overactive PI3K/AKT/mTOR signaling pathway, and upregulated macrophage polarization from M2 to M1 macrophages. It was determined that the results offered a translational approach to treating cisplatin-resistant OC and might provide a treatment option for other cisplatin-resistant tumors.

### 6.2 Hydrogels

The polymer chains of a hydrophilic material are cross-linked to form a three-dimensional (3-D) network called a hydrogel. These materials show great promise for a wide range of biomedical applications because they are simple to fabricate, biocompatible, can be tuned to achieve the desired composition, and have superior physical properties. The main goal of hydrogel-based technology is the creation of injectable hydrogels, in which the gel precursor, typically aqueous, is combined with other biologically active substances or biopolymers before being injected into the desired area of interest using a syringe (Jiang et al., 2022; Xin and Naficy, 2022). The primary benefit of injectable hydrogels is their highly adaptable characteristics, which, when applied *in vivo*, result in a rapid recovery with a smaller scar size and minimal pain for the patients, while retaining a higher capacity and a more efficient drug or gene encapsulation for their delivery. Injectable hydrogels that undergo *in situ* gelation are superior for targeted and localized drug delivery inside tumor cells because they can both keep their contents safe inside the tumor and incisively releases the drugs into cancerous cells (Sheng et al., 2017).

**FIGURE 6:**
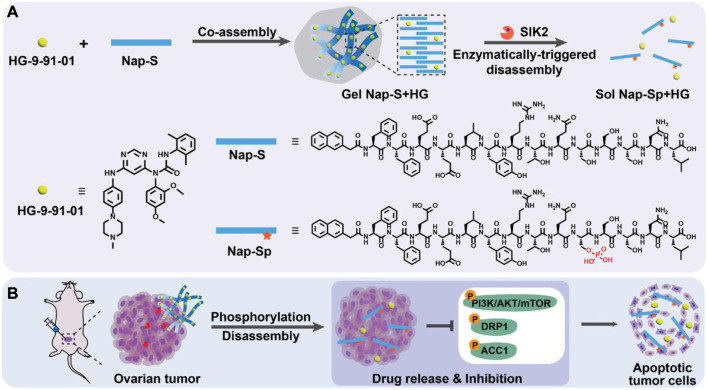
Chemicals and conceptual illustrations are included in this work. **(A)** Gel Nap-S + HG is formed and then disassembled to release HG *via* SIK2-mediated Nap-S phosphorylation to Nap-Sp, as shown schematically. **(B)** OC cells can be effectively induced to undergo apoptosis *via* inhibition of SIK2 downstream protein phosphorylation, which can be achieved through Gel Nap-S + HG local delivery for SIK2-responsive and sustained HG release (Hua et al., 2022).

Due to its essential function in progression and tumorigenesis, the salt-inducible kinase 2 (SIK2) is a highly attractive therapeutic target for OC. The clinical uses of SIK2 inhibitors are restricted due to the potential for significant off-target effects when administered directly. Hua et al. (2022) described a targeted nano-hydrogel for SIK2 inhibitor local administration to effectively impede the metastasis of OC. The desing and application strategy of this SIK2-responsive supramolecular hydrogel system is illustrated in Figure 6. By blocking SIK2 and the phosphorylation of its downstream signaling molecules, nano-hydrogels have been shown in cell experiments to have a profound therapeutic effect on cancer cells. Using an ovarian tumor model, researchers found that mice treated with a nano-hydrogel containing a SIK2 inhibitor significantly reduced tumor development and metastasis in comparison to mice given a free SIK2 inhibitor.

To deliver cisplatin to OC cells specifically and effectively and to enhance the drug’s anti-cancer effects, and prevent cisplatin resistance, Serini et al. (2021) created a hydrogel based on hyaluronic acid and folic acid. The drug’s gradual and controlled release from the polymeric network and its degree of swelling at physiological pH indicated that it was suitable for the delivery of cisplatin in OC. In addition to inhibiting OC cell growth and migration more effectively than pure cisplatin, cisplatin-loaded hydrogel also altered proteins expression during the Epithelial-Mesenchymal Transition, a crucial process in the development of OC’s resistance to cisplatin and metastatic spread.

### 6.3 Polymeric micelles

Polymeric micelles are nanostructures that resemble the morphology of dendrites and liposomes. The outer layer is typically composed of hydrophilic polymers, while the inner core contains drugs that are poorly soluble in water and is hydrophobic. The polymeric micellar’s outer shell serves as the structure’s barrier against interactions with the reticuloendothelial system and blood constituents. The hydrophilic exterior can be modified by polyethers such as poly (ethylene oxide) and PEG. Covalent bonding, hydrogen bonding, π-π interactions, electrostatic bonding, and hydrophobic interactions are some of the cohesive interactions between the micelle’s inner core and the entrapped drug (Bhardwaj et al., 2022).

**FIGURE 7:**
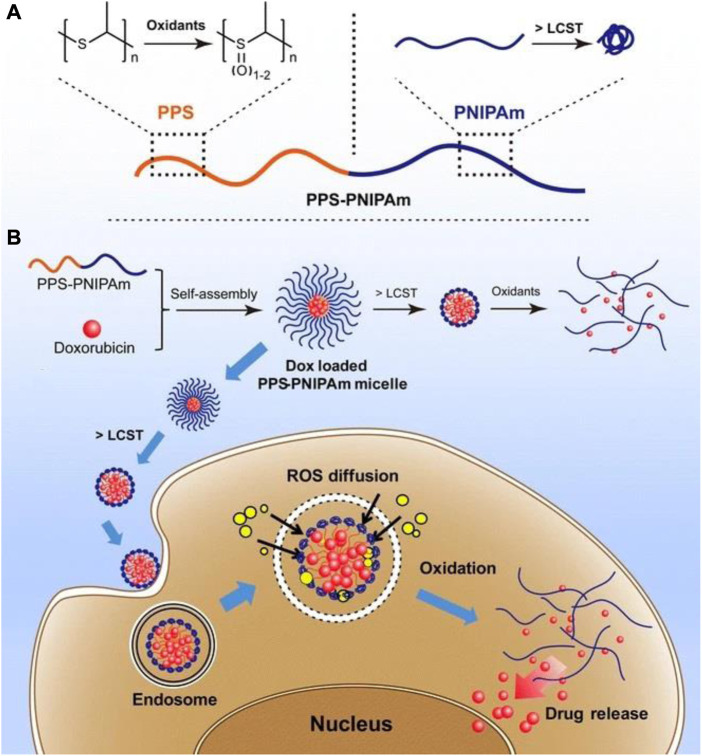
Representation of the stimuli-response of the block copolymer PPS-PNIPAm. **(A)** The PPS block’s oxidation responsiveness and the PNIPAm block’s temperature responsiveness. **(B)** Self-assembly of PPS-PNIPAm micelles and doxorubicin loading. To further oxidize and liberate enclosed DOX molecules, the PNIPAm corona must first collapse and shrink over its LCST and PPS core. This type of drug-loaded nanocarrier can be exploited for cancer cell transport and drug release in an intracellular ROS environment (Tang et al., 2017).

In the treatment of OC, nano-micelles are promising nanocarriers due to their special properties, which include tumor perforation, hydrophobic chemotherapeutic loading, high biocompatibility, prolonged circulation in plasma, and *in vivo* stability (Shariatinia, 2021). Redox-sensitive nano micelles containing paclitaxel were created for the treatment of chemotherapy-resistant OC (Mutlu-Agardan et al., 2020). Such a micellar nanosystem was utilized to treat OC cells SKOV-3 in a redox-sensitive manner. Docetaxel’s folate-targeted nanomicelles were also fabricated and tested in SKOV3 OC cell lines for pharmacokinetic and cytotoxic effects (Kazemi et al., 2021). According to the findings, the cytotoxicity of the micellar system with docetaxel was significantly higher than that of free drugs. Recently, a method was reported for fabricating polymeric micelles to determine the high loading capacity of two hydrophobic drugs, doxorubicin, and irinotecan (Wu et al., 2020). The drug-loaded micelles demonstrated drug high ultra loading, desired size distribution, significant uptake of OC cells, effective biocompatibility, profound stability, and, most importantly, overproduction ROS that led to effective cargo release in the environment of cancer cells. Furthermore, *in vitro* and *in vivo* studies confirmed that the micellar system significantly stimulated anti-OC activity. A thermally and reactive oxygen species-responsive nanocarrier system comprised of PPS-PNIPAm block copolymer for cancer treatment was developed byTang et al. (2017). The PPS block was used to make the system oxidation responsive while the PNIPAm block was employed for temperature responsiveness. Doxorubicin was loaded in the self-assembled PPS-PNIPAm micelles. The release mechanism and potential application of this system is shown in Figure 7.

### 6.4 Solid-lipid nanoparticles

**FIGURE 8:**
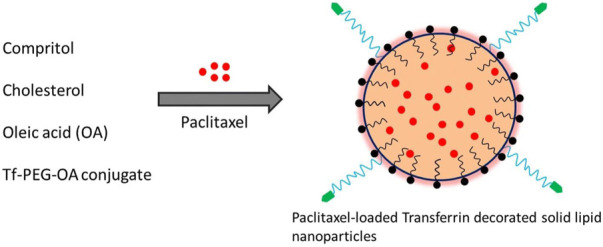
Schematic depiction of the synthesis of PTX-loaded, transferrin-coated SLNs (Dai et al., 2018).

Solid-lipid nanoparticles (SLNs) are spherical colloidal nanocarriers composed of lipids, surfactants, and chemotherapeutics in an appropriate ratio. Their average diameter is between 50 and 1,000 nm. SLNs are superior to currently available polymeric nanoparticle-based drug carriers due to their nano size and lipid core. Due to their long duration of circulation, notable biocompatibility, and superior tumor accumulation as a result of the EPR effect, lipid-based nanostructures have attracted considerable interest as drug nanocarriers (Bukhari et al., 2021; Loh et al., 2021). To produce an appropriate nanoformulation of paclitaxel for parenteral delivery, Lee et al. (2007) developed paclitaxel-encapsulated, sterically stabilized SLNs for the therapies of the breast cancer cell line MCF-7 and the human OC cell line OVCAR-3. The cytotoxicities of the SLNs-based paclitaxel-loaded system as created were comparable to those of a commercial paclitaxel formulation based on Cremophor EL, suggesting the possibility of the SLNs-based nanoplatforms as a novel delivery method for parenteral administration routes. A clinically approved photosensitizer verteporfin medication was loaded into nanostructured lipid carriers for OC treatment (Michy et al., 2019). Effective internalization of both free and lipid nanocarrier-loaded-verteporfin in ovarian carcinoma cells dramatically decreased the viability of tumor cells after laser light exposure. Analyses of pharmacokinetics and biodistribution revealed that lipid nanocarriers have a longer circulation duration and effective tumor uptake. Five of the eight tested mice died after receiving a 2 mg/kg dose of free verteporfin, whereas an intravenous injection of 8 mg/kg NLC-verteporfin significantly suppressed the growth of tumors without causing any obvious toxicity effects. Recently, Han et al. (2019) created SLNs loaded with paclitaxel for the intraperitoneal treatment of OC. Data showed that the cytotoxicity of paclitazel loaded solid lipid microparticles (PTX-SLMPs) was significantly increased as compared to Taxol^®^ in SKOV-3 OC cells. After administering PTX-SLMPs to Wistar rats, *in vivo* pharmacokinetic analysis showed a slow PTX absorption into the circulation, indicating that PTX-SLMPs were retained in the peritoneal cavity for a longer period. Studies have shown that solid-lipid-based nanocarriers can be utilized effectively in the targeted therapy of OC and can be expanded to other peritoneal malignancies. Dai and co-workers have reported transferrin-decorated paclitaxel (PTX)-loaded lipid nanoparticles (TPLN) for improving the chemotherapy response of cancer cells. The nanoparticles system was prepared by dissolving the drug (PTX), cholesterol and transferrin conjugated lipid (Tf-PEG-OA) in organic solvent and then evaporated the solvent through evaporation as shown in Figure 8. Their findings demonstrated the tf-conjugated lipid nanoparticle system’s ability to specifically target cancer cells, opening the door to effective cancer therapy (Dai et al., 2018).

### 6.5 Inorganic nanoparticles (INPs)

**FIGURE 9:**
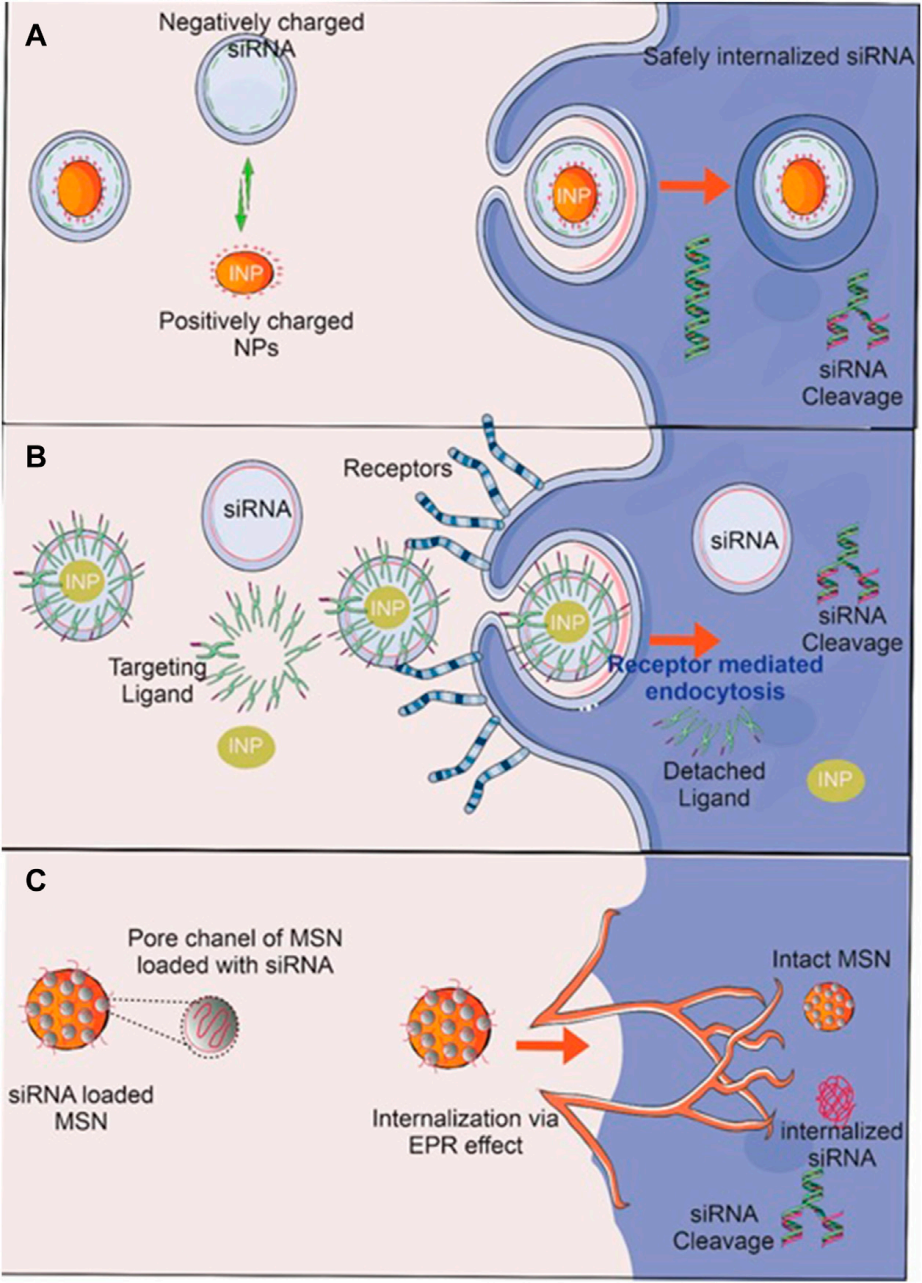
RNA delivery techniques using inorganic nanoparticles (INPs). Surface conjugation of INP by **(A)** siRNA *via* electrostatic attraction and **(B)** adsorption of siRNA delivery to cancer cells and a suitable ligand that targets corresponding receptors on cancer cells leads to the siRNA’s safe internalization, where it cleaves and suppresses oncogenes. **(C)** siRNA-impregnated MSN protects siRNA from degradation by interacting directly with enzymes, environmental pH, ions, and proteins, enabling its safe and efficient delivery to a target site *via* the enhanced permeation and retention (EPR) effect, where oncogenes are then cleaved and suppressed.

In recent years, the production of metal NPs, particularly iron, silver, gold, and metal oxide NPs, for the treatment of OC has attracted considerable attention. Metal NP synthesis and modification are influenced by their size, shape, and target specific accumulation for developing effective nanotechnology strategies. They improve permeability, reduced toxicity, and side effects while also improving the site-specific delivery of an anticancer drug. Additionally, it provides a large surface area and improves photosensitization for photothermal therapy. Moreover, Surface conjugation of drugs and siRNAs with INPs *via* electrostatic attraction, adsorption and infiltration **(** Figure 9 **)** make them suitable for targeting corresponding receptors on cancer cells. Bertucci et al. (2019) created OC-specific Micro RNA-silencing porous silicon NPs. It dramatically reduces tumor growth in xenografted tumor-bearing mice by suppressing miR-21. Brandhonneur et al. (2018) generated molybdenum octahedral cluster-loaded PLGA-NPs for OC photodynamic treatment. It was discovered that NPs had a negative zeta potential and were about 100 nm in size. The A2780 OC cell line was tested, and the formulation was found to be non-toxic at the concentrations utilized in the study. In comparison to non-activated settings, NPs were able to reduce cellular viability by up to 50%. It was determined that the formulation provided effective tumor targeting. Kotcherlakota et al. (2019) produced cationic AuNPs as well as gene (p53DNA) NPs. These NPs were resistant to breakdown by DNAse-I and exhibited serum stability. When the formulation was intraperitoneally delivered to a mouse model of SK-OV-3 cancer, it resulted in much-improved tumor targeting and tumor degradation. Wang and his colleagues produced doxorubicin-loaded silica nanoshuttles with magnetic and AuNP-embedded AuNP for the treatment of breast cancer (Wang et al., 2017). It was tested on *in-vitro* epithelial HeLa ovarian cells and demonstrated significantly increased cellular uptake and anticancer efficacy without toxicity. Chen and colleagues synthesized selenium/ruthenium NPs loaded with RNAs (siRNAs) that inhibited taxol-resistant MCF-7/T cells microtubule dynamics (Chen et al., 2017). The formulation facilitated siRNA intracellular uptake and promoted siRNA leakage from lysosomes/endosomes, resulting in the silencing of MDR genes in MCF-7/T cells. Apoptosis is promoted *via* MAPK, PI3K/Akt, and p-53 phosphorylation, which in turn increases cytotoxicity.

## 7 Nanotheranostics

**FIGURE 10:**
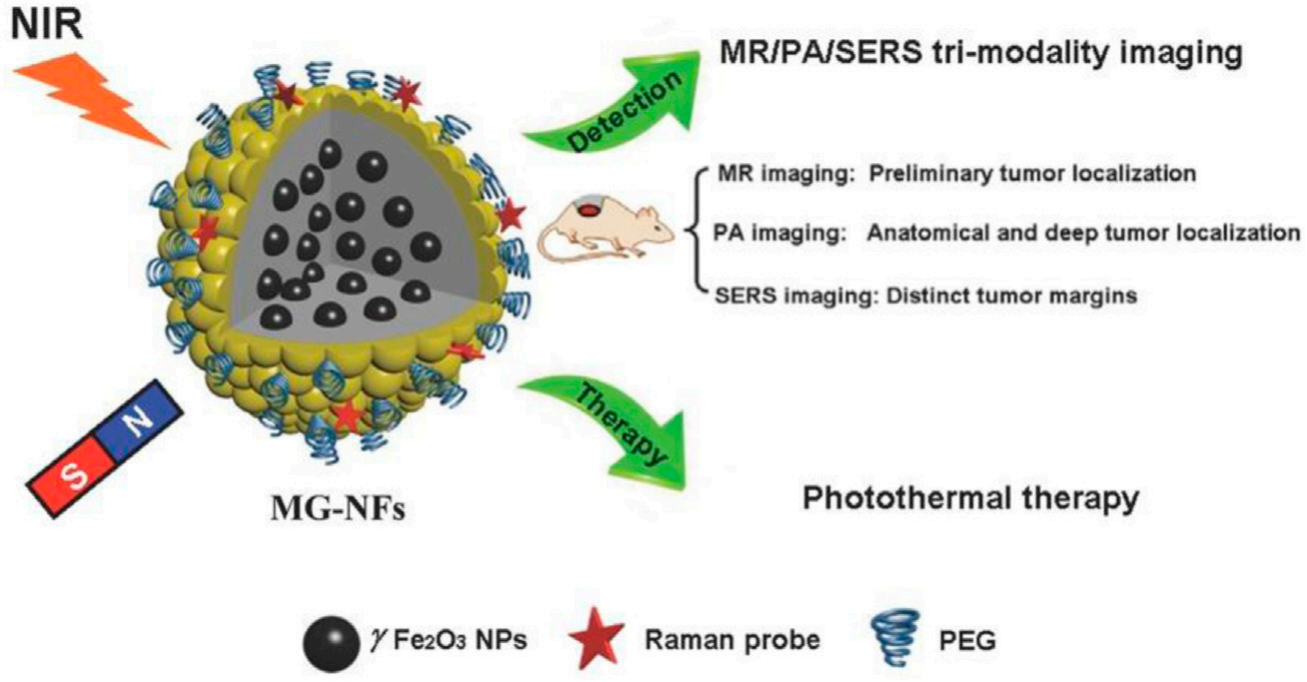
The illustration of magnetic gold nanoflowers (MG-NFs) with a core/shell of γFe_2_O_3_@Au for tumor photothermal therapy and multimodal imaging. Reproduced with permission from reference (Huang et al., 2015).

Theranostic nanoformulations are a combined method for the delivery of therapeutic drugs and the performance of a specific diagnosis. It describes the diverse medical and biomedical applications. Nanotheranstics carrier systems can be employed for a variety of personalized therapy functions, including initial disease diagnosis, disease stage, treatment planning, treatment selection, and identification of adverse or toxic effects. As a result of the nanoscale particle size, it possesses several advantages in treatment and diagnostics through the utilization of nanoformulation and nanosensors. A wide range of biomarkers can be detected by nanosensors in a small sample volume, and nanoformulations can deliver therapeutic agents from the bloodstream to the cancer site at a higher dose with fewer or no adverse effects (Liu et al., 2018). It offers a large surface area, prolonged drug release, and minimal immunogenicity. It had disadvantages like toxicity, non-biodegradability, poor stability, and quick removal from the body. PLGA-NPs loaded with molybdenum clusters were developed by Brandhonneur and its colleagues as a diagnostic and therapeutic option for OC (photodynamic therapy) (Brandhonneur et al., 2018). The formulation was evaluated on A2780 OC cells *in vitro*. In comparison to traditional formulations, it demonstrated a 50% reduction in cellular viability. Bogdanov and his colleagues generated AuNPs as theranostic (therapeutic and diagnostic) agents (Bogdanov et al., 2015). AuNPs stabilized by MPEG-gPLL displayed a significant increase in transmembrane permeation and significantly reduced endothelial cell toxicity. It showed increased cytotoxicity in a dose-dependent manner in epithelioid cancer cells. Edelman designed serum albumin and hyaluronic acid-based NPs containing fluorescein isothiocyanate and paclitaxel as a theranostic agent (Edelman et al., 2019). The formulation demonstrated selectively significant cytotoxicity and absorption toward CD44 expression by OC cells, in addition to diagnosing the site of the tumor. For the diagnosis and treatment of OC, Satpathy and its colleagues created magnetic Fe_2_O_3_-NPs conjugated with cisplatin and HER2 antibody. In nude mice, NPs significantly reduced tumor growth, increased cellular uptake, and induced cell apoptosis. Additionally, NPs were able to detect MRI due to HER2 expression on OC cells (Satpathy et al., 2019). Huang and his co-workers designed GSH-sensitive-Pt-containing hybrid lipid polymeric NPs as a theranostic for OC. By increasing ROS with few side effects, the formulation demonstrated improved therapeutic effectiveness and decreased chemotherapeutic toxicity (Huang et al., 2019). Magnetic gold nanoflowers (MG-NFs) with an Au core/shell for cancer theranostics have been used in recent years. For magnetic resonance (MR)/PA imaging/Surface-enhanced Raman spectroscopy (SERS) multimodal imaging, SERS-guided surgery, and photothermal therapy of tumors, γFe2O3@Au core/shell type MG-NFs and their application are given in Figure 10. Even at modest Au concentrations, prepared MG-NFs display impressive SERS enhancement, strong PA signals, enhanced relaxivity, and efficient photothermal effect.

## 8 Conclusion and outlook

Due to the effectiveness of nanotechnology based drugs delivery vehicles, it is gaining much attention for effective cancer treatment. In this review, the currently investigated nanocarriers for controlled drug delivery are discussed as a developing strategy in cancer therapy, along with the need for the development of such strategies in terms of targeted therapy. Several nanocarriers, including polymeric systems, polymeric micelles, liposomes, inorganic NPs, and hydrogels have been thoroughly investigated for the delivery of various chemotherapeutics for various cancer treatments. As a result, an emerging strategy for the treatment of OC in the future could involve the administration of multiple chemotherapeutic drugs in a suitable nanocarrier, either in combination or individually. Nanocarriers' safety, administration route, ease of delivery, *in vitro* and *in vivo* efficacy, and stability are some of the factors that need to be taken into account, along with their physicochemical characteristics, materials, and loaded cargo.

One of the most advanced and encouraging methods of treating OC is nanomedicine. Numerous studies have indicated that the use of nanomedicine therapeutics for the treatment of OC is highly effective both *in vitro* and *in vivo*. However, there are currently only a small number of nanocarrier-based drugs that have been approved for clinical use. Regulatory issues, safety concerns, nanomedicines' physicochemical characterizations (size, shape, drug loading, surface distribution, biodegradability, surface chemistry, etc.), and manufacturing problems are some of the obstacles that must be overcome in the clinical application of these nanomedicines. In addition, research that is based on polymers will undoubtedly continue to thrive, and scientists have to focus their efforts on further designing and modifying polymers to address photobleaching issues and a short blood circulation life in practical applications.

Future advances at the interface of physical sciences and engineering will contribute to the development of innovative approaches that will provide patients and physicians with the information, therapeutics, and diagnostics necessary to eradicate diseases, such as OC. Over the past 5 decades, the combined effects of the fields of engineering, physical sciences, and oncology has proven to be an effective strategy for the treatment of OC, resulting in a technological and medical revolution. The integration of these fields also has the potential to speed up the diagnosis of OC at an extremely early stage, which will prevent the need for costly, invasive treatments for metastatic cancer at a late stage. The effectiveness of any treatment can be further increased by employing synergistic strategies such as immunotherapy and chemotherapy to boost immune system to fight cancer. Nanotechnology based devices and implants can be directly placed in tumor sites through minimally invasive surgery that will increase the *in vivo* effectiveness of chemotherapy and make it more cost-effective.
